# Managing the Leeway Space in Mixed Dentition Using a Passive Lingual Arch: A Systematic Review

**DOI:** 10.3390/dj13030135

**Published:** 2025-03-20

**Authors:** Alberto De Stefani, Giovanni Bruno, Valentina Montanari, Ayoub Boutarbouche, Patrizio Bollero, Antonio Gracco, Michele Basilicata

**Affiliations:** 1 Department of Neuroscience, School of Dentistry, University of Padova, 35121 Padova, Italy; alberto.destefani@unipd.it (A.D.S.); valentina.montanari93@gmail.com (V.M.); ay.boutarbouche@gmail.com (A.B.); antonio.gracco@unipd.it (A.G.); 2 Department of Pharmacological Sciences, University of Padova, 35121 Padova, Italy; 3 Department of Industrial Engineering, University of Rome Tor Vergata, 00133 Rome, Italy; 4 Department of System Medicine, University of Rome Tor Vergata, 00133 Rome, Italy; patrizio.bollero@ptvonline.it; 5 UOSD Special Care Dentistry, Department of Experimental Medicine and Surgery, University of Rome Tor Vergata, 00133 Rome, Italy; michele.basilicata@ptvonline.it; 6 UniCamillus-Saint Camillus International University of Health Sciences, 00133 Rome, Italy

**Keywords:** dental crowding, premature loss of deciduous elements, passive lingual arch, lower arch length, leeway space

## Abstract

**Background/Objectives:** Dental crowding and the premature loss of one or more deciduous teeth are common issues during the growth phase that accompanies the transition from mixed to permanent dentition. The aim of this systematic review is to examine the effectiveness of using a passive lingual arch in preserving the length of the lower arch and managing the leeway space, analyzing the effects on the linear and angular positions of the permanent teeth. **Methods:** A systematic review of the literature was conducted using the PubMed, Web of Science, Scopus, and Cochrane Library database. After an initial selection of 306 articles, seven studies that met the defined selection criteria were included. These articles were used to compile the PICO table. **Results:** The studies examined agree that the application of the passive lingual arch is useful in preserving the length of the lower arch during the transition from mixed to permanent dentition. The observed changes in the linear and angular positions of the permanent teeth, particularly the distoinclination of the permanent molars and the proclination of the incisors, were considered indicative of the effectiveness of this technique. However, one author did not observe these changes, noting only a prevention of mesioinclination and lingualization of the molars and incisors. **Conclusions:** The use of the passive lingual arch in the transition from mixed to permanent dentition proves to be advantageous for correcting mild anterior crowding, maintaining residual spaces after the premature loss of deciduous molars, and preventing the impaction of permanent premolars. This simple and effective orthodontic device can be applied in clinical practice, always based on an accurate diagnosis and a well-defined treatment plan.

## 1. Introduction

The leeway space (LWS) represents a crucial physiological discrepancy in the transition from mixed to permanent dentition. It is defined as the mesial–distal width difference between the second deciduous molars and the second permanent premolars, averaging 2.5 mm per hemiarch in the lower arch and 1.5 mm in the upper arch, constituting an essential resource for dental alignment, particularly in cases of mild or moderate anterior crowding [1]. Proper utilization of this space through targeted orthodontic interventions allows for the preservation of arch length and the optimization of the perimeter necessary for the eruption of subsequent permanent teeth [1].

Maintaining the LWS is particularly relevant in cases of premature loss of deciduous teeth, a condition that facilitates the mesial migration of permanent molars, thus reducing the available space for permanent canines and premolars [2,3]. The passive lingual arch is one of the most effective orthodontic devices for managing this spatial resource, thanks to its ability to prevent the undesired movement of erupted permanent teeth and to maintain the space intended for developing elements [4]. This device is associated with significant changes in dental position, including the proclination of incisors and the distoinclination of molars, which promote harmony in the lower arch.

Premature loss of deciduous elements, caused by factors such as trauma or carious lesions, is a common issue that requires orthodontic intervention, as it leads to a reduction in arch perimeter and an alteration in the sequence of dental eruption [5]. Dental crowding is one of the most frequent malocclusions in mixed and permanent dentition, with a prevalence reaching 50% in children between 8 and 11 years old, increasing further in permanent dentition [6]. Such crowding can be classified as primary, due to structural discrepancies; secondary, associated with environmental factors such as carious lesions; and tertiary, which emerges during adolescence in relation to late mandibular growth. Its management varies from conservative interventions for mild or moderate crowding to more invasive procedures, such as serial extractions, for severe or extreme crowding [6].

**Figure 1:**
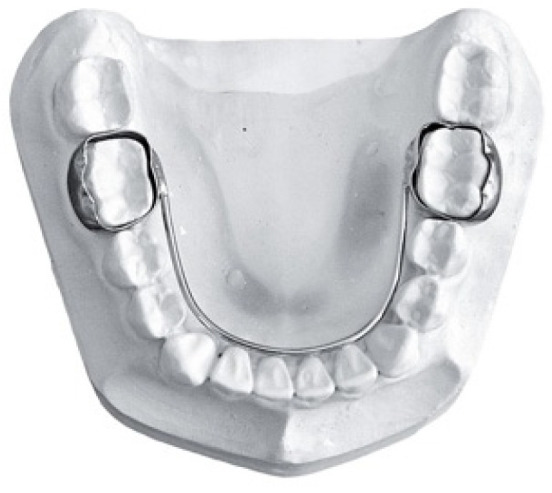
Example of passive lingual arch.

The use of orthodontic devices plays a central role in managing dental space. Space maintainers, such as the band and loop maintainer or the passive lingual arch (PLA) (Figure 1), are employed to prevent the migration of permanent molars [7], while active devices, such as the lip bumper or the active lingual arch, are designed to recover space through controlled dental movements [8]. In cases where the available space is insufficient, the lip bumper, generating a distalizing force on mesially inclined molars, and the active lingual arch, capable of proclining the incisors and distalizing the molars, prove to be effective tools for significant space recovery [8].

When crowding exceeds 7 mm, a planned sequence of serial extractions is necessary, which includes the extraction of deciduous canines to facilitate the alignment of the incisors, followed by the extraction of the first deciduous molars, and finally, the first permanent premolars to ensure the correct eruption of canines. However, the choice of treatment must be based on an accurate assessment of the severity of crowding, the patient’s age, and the potential for residual growth, favoring a conservative approach in mild or moderate cases and reserving complex orthodontic treatments for more severe cases [9,10,11,12,13,14].

The PLA specifically addresses several orthodontic concerns effectively. As a non-removable, fixed orthodontic appliance, the PLA stand across the lingual side of the lower molars, secured by bands cemented around the molars. This device plays a crucial role in utilizing the natural leeway space created during the transition from deciduous to permanent dentition, allowing for the strategic management of space without the active cooperation of the patient. The PLA works by preventing mesial drift or undesired shifting of the molars. This stabilization is especially critical in preventing the closure of the dental arch, thus avoiding complications associated with crowding and misalignment.

The management of space in mixed dentition is a critical aspect with significant implications in growing patients. The passive PLA is widely used to preserve lower arch length and for the management of the leeway space by preventing the mesial migration of permanent molars and the loss of space for canines and premolars. However, despite its widespread clinical application, there is ongoing debate regarding the actual effectiveness of the PLA compared to other treatment options and its long-term impact on dental positioning.

This systematic review aims to examine the effectiveness of using the PLA in managing the LWS during the transition from mixed to permanent dentition. The goal is to analyze changes in the length of the lower arch, and the linear and angular positions of the permanent teeth, thus contributing to evaluating the clinical utility of this device for the treatment of dental crowding and for maintaining space in the arch.

## 2. Materials and Methods

### 2.1. Information Sources

This systematic literature review was conducted using specialized scientific databases, including PubMed, Web of Science, Scopus, known for the quality and relevance of the articles indexed in the fields of dentistry and orthodontics. These sources were selected to ensure comprehensive coverage of the available literature, including both primary studies and theoretical references that define the methodological and practical bases for strategies to manage dental arch space during mixed dentition. Cochrane Library was also used to check if previous systematic reviews have been published on the field.

This systematic review was conducted in accordance with the PRISMA (Preferred Reporting Items for Systematic Reviews and Meta-Analyses) guidelines and checklist. This review was registered on PROSPERO database (CRD420251000668).

### 2.2. Search Strategy

The literature search was conducted over the period from October 2022 to February 2025 using combinations of keywords, selected based on terminology most frequently employed in the specialist literature. Complex search strings were used, combined through Boolean operators (AND, OR) and supplemented with the use of filters to ensure a targeted selection of the most relevant articles. The main search strings included:

- (“Lee way” OR “lee way” OR “Leeway” OR “leeway” OR “Leeway” OR “leeway” OR “E space” OR “e space” OR “E-space” OR “e-space”) AND (“management” OR “use”)
- (“Lee way” OR “lee way” OR “Leeway” OR “leeway” OR “Leeway” OR “leeway” OR “E space” OR “e space” OR “E-space” OR “e-space”) AND (“crowding”)
- (“Lee way” OR “lee way” OR “Leeway” OR “leeway” OR “Leeway” OR “leeway” OR “E space” OR “e space” OR “E-space” OR “e-space”) AND (“management” OR “use” OR “distribution”) AND (“crowding”)
- (“Leeway space” OR “leeway space” OR “Leeway space” OR “leeway space” OR “E space” OR “e space” OR “E-space” OR “e-space”) AND (“tooth” OR “teeth”)
- (“Leeway space” OR “leeway space” OR “Leeway space” OR “leeway space” OR “E space” OR “e space” OR “E-space” OR “e-space”)
- arch length preservation
- passive lingual arch

The search was limited to studies published between 1972 and February 2025.

### 2.3. Screeening Process

The inclusion and exclusion criteria were established based on population, interventions, comparisons, outcomes, and study designs (PICO guidelines). The selection process followed a multi-stage approach: Initially, titles were examined to eliminate duplicates and clearly irrelevant articles. Subsequently, the abstracts of potentially eligible studies were analyzed to identify those that met the research objectives. Authors included studies published between 1972 and February 2025 with available abstracts and full texts. Authors included retrospective studies, cross-sectional studies, prospective studies, and randomized controlled trials and excluded literature reviews, case reports, and articles with incomplete or non-standardized methodologies. Articles deemed relevant were read in full and analyzed using the PICO (Patient, Intervention, Comparison, Outcome) methodology to standardize data extraction and ensure accuracy in evidence evaluation.

### 2.4. Methodological Quality Criteria

Methodological quality criteria was adapted from the CONSORT statement and evaluated design of the randomized clinical trial, eligibility criteria for study participants, sample size determination, details about clinical diagnostic criteria, ethical considerations, method of blinding, methods and type of randomization, description of recruitment period and follow-up, withdrawals and dropouts, clearly defined outcomes, and appropriate statistical analysis (Table 1).

## 3. Results

### 3.1. Study Selection

In this literature review, a total of 324 articles were identified through searches in electronic databases. Mendeley reference manager software (version 1.19.8) was used to collect the data. No additional articles were found from other sources. After removing duplicates (n = 18), 306 articles were screened based on title and abstract. Of these, 283 were excluded as they were not relevant to the objectives of the review. Subsequently, 23 articles were selected for full-text reading.

After further evaluation, 16 articles were excluded for the following reasons:

- Use of orthodontic devices other than the passive lingual arch (n = 4).
- Early loss of the first deciduous molar (n = 2).
- Use of a removable lingual arch (n = 3).
- Use of different orthodontic devices for managing the leeway space (n = 7).

**Figure 2:**
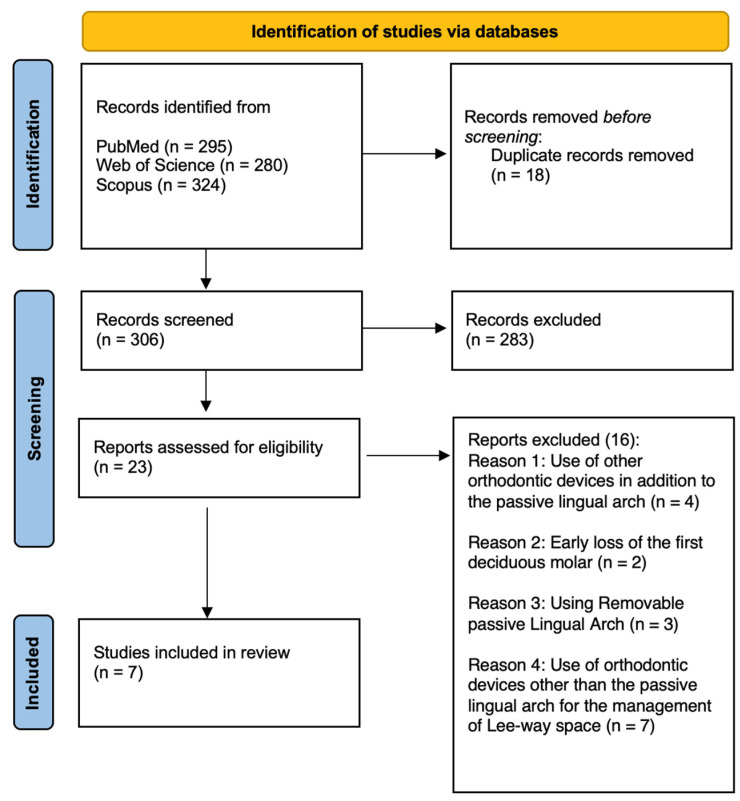
PRISMA selection of the included studies.

At the end of the process, seven studies were included in the systematic review (Figure 2).

### 3.2. Characteristics of Included Studies

The included studies were published between 1972 and February 2025. All articles analyzed the effectiveness of the passive lingual arch in managing leeway space and its effects on the lower arch, from mixed to permanent dentition. The main characteristics of the studies are summarized in Table 1:

- Sample: The sample size ranged from 20 to 150 patients per study, with an average starting age of 6 to 10 years.
- Duration of follow-up: The duration of treatment varied from 12 to 36 months.
- Measured variables:
  ○ Changes in arch length.
  ○ Stability of the alignment of the lower anterior teeth.
  ○ Conservation of leeway space.
- Orthodontic device used: All studies exclusively considered for the intervention the fixed passive lingual arch.

### 3.3. Data Extraction

Data were extracted according to the PICO (Patient, Intervention, Comparison, Outcome) methodology. In the patient section, we identified the study population and the control group. In the intervention section, we identified the treatment performed on the study sample. In the comparison section we identified what the authors compared in the study sample (position of teeth, study models, and cephalograms). In the outcome section, we identified the main outcomes of the study, including inter-canine width, intermolar width, arch length, molars inclination, incisors inclination, and vertical, sagittal, and transversal movements, were extracted from the selected articles.

Rebellato et al. [4] found that, in the control group, the molar tipped forward 2.19° and the cusp tip came forward 1.73 mm. The measurements for the treatment group were −0.54° (backward tip), and 0.29 mm, respectively (*p* < 0.001). In the control group, the incisor angulation change was −2.28° (backward tip) and the incisal edge was reduced by 0.65 mm. The data for the treatment group indicated 0.73° of forward tip of the incisor and 0.44 mm advancement of the incisal edge (*p* < 0.0001).

Villalobos et al. [15] found that the PLA group reflects a minimal mesial drift of 0.15 ± 0.67 mm, a backward tip of −0.54° ± 1.78°, and a minimal extrusion of 0.29 ± 0.48 mm.

Fichera et al. [16] found a statistically significant differences between the mandibular posterior rotation (MPR) group and the other two groups: mandibular growth in straight-downward direction (MSD) and mandibular anterior rotation (MAR) were found as regards to the mandibular first molar and incisor positional changes. No significant differences were found between the MSD and MAR groups.

Owais et al. [17] found a proclination of lower incisors and space loss of the lower primary second molars. The group with the 0.9 mm PLA was superior to that made of 1.25 mm in terms of arch length preservation.

Joose et al. [18] found a similar mesial movement of the lower molar cusp between the PLA and no-PLA groups, but the vertical position was slightly greater, at T2 in the PLA group. In the PLA group, there was a molar tip-back effect, and the lower incisors were proclined 4.2° more than in the no-PLA group. Arch perimeter decreased 3.6 ± 2.6 mm without a PLA and 0.97 ± 3.7 mm with a PLA. Inter-canine and intermolar widths both increased about 1 mm more with a PLA (*p* < 0.0001)

Brennan et al. [19] found that the arch length decreased only 0.44 mm whereas the inter-canine, inter-premolar, and intermolar dimensions increased between 0.72 and 2.27 mm. There was adequate space to resolve the crowding in 65 (60%) of the 107 patients.

Singer [20] found that PLA causes tipping distal of incisors, positioning delays, and prevents tooth overlap. All the characteristics of the included studies are summarized in Table 2.

Methodological quality of the selected studies and the assessment of bias risk are reported in Table 3 and Table 4.

## 4. Discussion

During this literature review, some limitations were encountered in the search for studies to consider. Indeed, there are not many scientific studies on the management of leeway space in mixed dentition useful for resolving anterior crowding.

The articles reviewed mostly concerned the use of the passive lingual arch, which is why the research for this thesis was directed towards the use of this orthodontic device for managing leeway space. All the literature considered in this work agrees that the use of a passive lingual arch in the transition from mixed to permanent dentition allows for optimal management of the leeway space and thus preserves the length and perimeter of the lower arch. Some authors [3,15,16,17,18,19,20], however, noted that the preservation of the arch perimeter is made possible by some changes that occur within it, such as the physiological increase in inter-canine distance, but especially a significant proclination of the lower incisors, contrary to what would normally occur without the use of a passive lingual arch.

Conversely, Villalobos et al., in their study, did not demonstrate proclination of the incisors with the use of the passive lingual arch but showed how such a device can prevent the normal uprighting of the same incisors [15]. This difference in study results, however, can be explained by the choice of the sample selected in the research. Indeed, Fichera et al. observed how the direction of mandibular growth (antero-rotation, postero-rotation, or vertical direction) affects the degree of final proclination of the incisors; in particular, they demonstrated that patients with a postero-rotation growth direction showed greater proclination of the incisors compared to other individuals [16].

It is important to consider the changes associated with the use of this device during treatment planning. In patients with postero-rotation mandibular growth, the distoinclination of the first molar might accentuate dolichofacial characteristics, increasing the posterior vertical dimension. Conversely, this movement can be exploited to the advantage of the clinician in patients with antero-rotation mandibular growth or brachyfacial characteristics.

Regarding the changes observed in the first permanent molars, most of the literature has highlighted a tip-back of these dental elements, contrary to the mesioinclination they would normally undergo at the expense of the leeway space [3,15,18,20]. From these studies, it also emerged that the amount of distoinclination is directly proportional to the observation time. Conversely, the study by Owais et al. did not show significant differences regarding the angulation of the first permanent molars [17].

Another parameter investigated in the literature concerns the extrusion of the first permanent molars: the studies by Villalobos et al. [15] and Singer [20] have shown how the use of a passive lingual arch allows for vertical control at the molar level; conversely, Rebellato et al. [4] and Joosse et al. [18] have observed that this does not occur. Joosse et al., however, explained this lack of vertical control not so much with the extrusion of the dental element itself, but as a consequence of the distoinclination of the tooth, which leads the mesio-buccal cusp to assume a more occlusal position.

Finally, regarding the resolution of crowding, the study that investigated this the most was that of Brennan et al. [19], which demonstrated how the use of a passive lingual arch leads to a resolution of anterior crowding in 60% of patients, as such a device allows for proper management of the leeway space, leading to the conservation of 4.85 mm within the lower arch.

The expected outcomes of using a PLA in managing the leeway space include the preservation of lower arch length, the prevention of mesial migration of permanent molars, and the resolution of mild to moderate anterior crowding through optimal space maintenance. Furthermore, the observed changes in incisor proclination and molar distoinclination, while varying among studies, indicate that the PLA contributes to a more favorable dental alignment by strategically utilizing the available space. These effects, alongside its role in vertical control, support its clinical utility as a predictable and effective intervention in mixed dentition.

The present review also presents some limitations that must be acknowledged to evaluate the findings accurately. Firstly, the heterogeneity of samples across the included studies regarding age, ethnicity, and dentoskeletal characteristics could influence outcomes differently. Secondly, the limited duration of follow-up in some studies restricts our understanding of the long-term effects of the PLA. Additionally, the variation in measurement methods across studies, which include cephalometric, digital models, and manual overlays, introduces potential biases in data collection and result interpretation. Future research should focus on long-term prospective studies with standardized methodologies to better assess the stability of outcomes associated with the use of the passive lingual arch. Further investigations are needed to explore the influence of individual dentoskeletal characteristics on treatment response, as well as to compare the PLA with alternative space management strategies. From a clinical perspective, these findings reinforce the importance of an individualized treatment approach, where the selection of the PLA should be based on a comprehensive assessment of mandibular growth patterns, arch perimeter needs, and potential long-term effects on dental positioning. These limitations suggest that, while the study provides valuable insights, its conclusions should be interpreted with caution and an understanding of the potential for unaccounted variables influencing the outcomes.

## 5. Conclusions

The use of a passive lingual arch in the lower arch during the transition from a phase of mixed dentition to permanent dentition is a simple and effective solution to preserve the leeway space. This way, with a minimally invasive orthodontic appliance that carries minimal risks during treatment, it is possible to resolve a degree of anterior crowding of approximately 4 mm. If a premature loss of one or more deciduous molars occurs, there is a mesial migration of the first permanent molar and a lingualization of the incisors. These conditions lead to a reduction in the arch perimeter with a consequent aggravation of an already present crowding, the development of crowding if not already present, or eruptive difficulties of the permanent premolar. With the use of a passive lingual arch, however, the literature has shown that the length of the lower arch is easily maintained thanks to a distoinclination of the first permanent molars and a simultaneous proclination of the incisors. The literature agrees that the passive lingual arch is a useful tool in cases of mild to moderate crowding. Its use requires careful diagnostic phase and precise therapeutic planning.

## Figures and Tables

**Table 1:** Methodological quality criteria.

	ITEMS	SCORING
A	Design of randomized clinical trial	1
B	Eligibility criteria for study participants	1
C	Sample size determination	1
D	Details about clinical diagnostic criteria	1
E	Ethical considerations	1
F	Method of blinding	1
G	Methods and type of randomization	1
H	Description of recruitment period and follow-up	1
I	Withdrawals and dropouts	1
J	Clearly defined outcomes	1
K	Appropriate statistical analysis	1
	Total score	11

**Table 2:** Characteristics of the included studies.

Author	Year	Patient	Intervention	Comparison	Outcome
Singer [20]	1972	36 (16 M, 20 F) Control group of 17 untreated (9 M, 8 F)	PLA	Changes in the position of the first permanent molars and incisors with the use of PLA	PLA causes tipping distal of incisors, positioning delays, and prevents tooth overlap. Intercanine width: +0.48 mm Intermolar width: +2.08 mm Arch length: +0.48 mm
Fichera et al. [16]	2010	42 (20 M, 22 F, mean age 9 ± 0.8) divided in 3 groups according to the Gonial angle. Control group of 18 patients (8 M, 10 F mean age 9.2 ± 0.6)	PLA	Lateral cephalograms and study models before and after treatment	Statistically significant differences between the mandibular posterior rotation (MPR) group and the other 2 groups: mandibular growth in straight-downward direction (MSD) and mandibular anterior rotation (MAR) were found as regards mandibular first molar and incisor positional changes. No significant differences were found between the MSD and MAR groups. Arch length increased of 0.04 mm Intercanine width increased of 1.2 mm
Owais et al. [17]	2010	44 (24 M, 20 F, mean age 10.76. Control group of 23 (15 M, 8 F, mean age 10.6)	PLA of 0.9 mm and 1.25 mm stainless steel to maintain arch length	Lateral cephalograms and study models before and after treatment.	Proclination of lower incisors and space loss of the lower primary second molars. The group with the 0.9 mm PLA was superior to that made of 1.25 mm in terms of arch length preservation
Joosse et al. [18]	2022	98, mean age 8.5 ± 1.3. Control group of 39, mean age 8.3 ± 0.7	PLA	Changes in the position of permanent lower molars and incisors.	Mesial movement of the lower molar cusp was similar between the PLA and no-PLA groups, but the vertical position was slightly greater at T2 in the PLA group. In the PLA group, there was a molar tip-back effect, and the lower incisors were proclined 4.2° more than in the no-PLA group. Arch perimeter decreased 3.6 ± 2.6 mm without an PLA and 0.97 ± 3.7 mm with an PLA. Intercanine and intermolar widths both increased about 1 mm more with an PLA (*p* < 0.0001)
Villalobos et al. [15]	2000	23 (11 M, 12 F, mean age 10.4 ± 0.6) 24, mean age 10.6	PLA	Pre-treatment and post-treatment cephalograms were used to determine positional changes	Measurements for the PLA group reflect a minimal mesial drift of 0.15 ± 0.67 mm, a backward tip of −0.54° ± 1.78° and a minimal extrusion of 0.29 ± 0.48 mm. PLA is a useful tool to control the vertical development of the mandibular molars and preserving arch length
Brennan et al. [19]	2000	107 (43 M, 64 F, mean age 8.6 with a range of 7 to 11 years)	PLA	Measurement of arch length using PLA and possibility of avoiding early tooth extraction	Arch length decreased only 0.44 mm whereas the intercanine, interpremolar, and intermolar dimensions increased between 0.72 and 2.27 mm. There was adequate space to resolve the crowding in 65 (60%) of the 107 patients
Rebellato et al. [4]	1997	14, mean age 11.5 Control group of 16, mean age 11.3	PLA	Study models, cephalograms, and tomograms of the patients to compare changes in the position of permanent molars and incisors	In the control group, the molar tipped forward 2.19° and the cusp tip came forward 1.73 mm. The measurements for the treatment group were −0.54° (backward tip), and 0.29 mm, respectively. The differences were all found to be statistically significant (*p* < 0.001). In the control group, the incisor angulation change was −2.28° (backward tip) and the incisal edge was reduced by 0.65 mm. The data for the treatment group indicated 0.73° of forward tip of the incisor and 0.44 mm advancement of the incisal edge. These differences were all found to be statistically significant (*p* < 0.0001)

**Table 3:** Methodological quality of selected studies.

		Quality Criteria Items												
Authors	Year	A	B	C	D	E	F	G	H	I	J	K	Total Score	Quality
Brennan et al. [19]	2000	1	0	0	0	0.5	0	1	1	0	1	1	5.5	Sufficient
Villalobos et al. [15]	2000	1	0	0	0	0	0	1	1	0	1	1	5	Sufficient
Fichera et al. [16]	2011	1	0.5	0	0	0.5	0	1	1	0.5	1	1	6.5	Fair
Owais et al. [17]	2011	1	0	1	0	0.5	0	1	1	1	1	1	7.5	Fair
Rebellato et al. [4]	1997	1	0	1	0	0.5	0	1	1	0.5	1	1	7	Fair
Joosse et al. [18]	2022	1	0	0	0	0.5	0	1	1	0.5	1	1	6	Sufficient
Singer [20]	1972	1	0	0	0	1	0	1	1	0	1	1	6	Sufficient

**Table 4:** Assessment of bias risk.

Author	Year	RCT	Concealment of Allocation	Early RTC Termination	Blinding of Patients	Blinding of Caregivers	Blinding of Data Collectors	Blinding of Outcome Assessors	Overall Bias
Brennan et al. [19]	2000	No	No	No	No	No	No	No	High
Villalobos et al. [15]	2000	No	No	No	No	No	No	Yes	Moderate
Fichera et al. [16]	2011	No	No	No	No	No	No	Yes	Moderate
Owais et al. [17]	2011	Yes	No	No	No	No	No	Yes	Low
Rebellato et al. [4]	1997	Yes	Yes	No	No	No	No	Yes	Low
Joosse et al. [18]	2022	No	No	No	No	No	No	Yes	Moderate
Singer [20]	1972	No	No	No	No	No	No	Yes	Moderate

## Data Availability

Data available on request.
